# Effect of Thermal Radiation on Three-Dimensional Magnetized Rotating Flow of a Hybrid Nanofluid

**DOI:** 10.3390/nano12091566

**Published:** 2022-05-05

**Authors:** Adnan Asghar, Liaquat Ali Lund, Zahir Shah, Narcisa Vrinceanu, Wejdan Deebani, Meshal Shutaywi

**Affiliations:** 1 School of Quantitative Sciences, University Utara Malaysia, Sintok 06010, Malaysia; asgharadnan675@gmail.com; 2 KCAET Khairpur Mirs, Sindh Agriculture University, Tandojam Sindh 70060, Pakistan; liaquat_ali@ahsgs.uum.edu.my; 3 Department of Mathematical Sciences, University of Lakki Marwat, Lakki Marwat 28420, Pakistan; 4 Faculty of Engineering, Department of Industrial Machines and Equipments, “Lucian Blaga” University of Sibiu, 10 Victoriei Boulevard, 5500204 Sibiu, Romania; 5 Department of Mathematics, College of Science & Arts, King Abdulaziz University, Rabigh 21911, Saudi Arabia; wdeebani@kau.edu.sa (W.D.); mshutaywi@kau.edu.sa (M.S.)

**Keywords:** thermal radiation, hybrid nanofluid, Joule heating, radiation, dual solution

## Abstract

The effect of thermal radiation on the three-dimensional magnetized rotating flow of a hybrid nanofluid has been numerically investigated. Enhancing heat transmission is a contemporary engineering challenge in a range of sectors, including heat exchangers, electronics, chemical and biological reactors, and medical detectors. The main goal of the current study is to investigate the effect of magnetic parameter, solid volume fraction of copper, Eckert number, and radiation parameter on velocity and temperature distributions, and the consequence of solid volume fraction on declined skin friction and heat transfer against suction and a stretching/shrinking surface. A hybrid nanofluid is a contemporary type of nanofluid that is used to increase heat transfer performance. A linear similarity variable is–applied to convert the governing partial differential equations (PDEs) into corresponding ordinary differential equations (ODEs). Using the three-stage Labatto III-A method included in the MATLAB software’s bvp4c solver, the ODE system is solved numerically. In certain ranges of involved parameters, two solutions are received. The temperature profile $\theta \left(\eta \right)$ upsurges in both solutions with growing values of EC and Rd. Moreover, the conclusion is that solution duality exists when the suction parameter $S\geq S_{ci}$, while no flow of fluid is possible when $S<S_{ci}$. Finally, stability analysis has been performed and it has been found that only the first solution is the stable one between both solutions.

## 1. Introduction

Fluid dynamics study has attracted the interest of experts, scholars, and researchers from numerous fields in current times owing to its multiple applications in engineering, science, and technology, as well as biopolymer-based detectors for medical sensing applications and diagnosis. Sakiadis [1] initially suggested the notion of a boundary layer steady flow on a stretching two-dimensional surface. Later, Crane [2] modernized the thought of Sakiadis. Then he applied it on steady-flow two-dimensional linear exponentially stretching surfaces. He claimed that the speed with which a sheet is stretched from a slit is related to the distance between them. Due to the fact that nanoparticles can pass through tissues and cells, chemical researchers state that nanotechnology can offer innovative solutions for treating stenosis, which is harmful and can cause death. It is noteworthy to mention the advanced evolvement of nanoparticles in drugs [3,4,5,6,7]. Choi [8] initiated research with a focus on nanoparticles by revealing their dynamic and abnormal attributes. Furthermore, he identified a nanofluid as a mixture generated by spreading nanoparticles in a fluid. He defined nanomaterials as heat transfer dispersions with better thermal performance than base fluids or conventional liquids. Nanofluids are composed of tiny quantities of solid particles measuring 100 nm or less in size. Nanofluids can be developed by evenly spread nanoparticles in a conventional liquid. Nanofluids often incorporate nanoparticles with unique chemical and physical properties, for instance, carbides, oxides, carbon nanotubes, and metals [9]. Water, organic liquids, polymeric solutions, oils, and other common fluids are examples of base fluids [10]. It has been discovered that nanofluids have better thermal efficiency than their basic fluids. Consequently, nanofluids have abundant real-world applications, including heat transfer. Agriculture, refrigerators, aerospace, and automobiles are just a few examples of their applications [11]. Khan and Pop [12] examined numerically the stretched surface in a nanofluid with a steady two-dimensional flow of the boundary layer. Miklavčič and Wang [13] are credited with being the first to explore the viscous steady three-dimensional flow across a shrinking surface using suction. Bachok et al. [14] examined a nanofluid with an unsteady two-dimensional boundary layer flow on a stretched/shrunk sheet. Moreover, Deroet al. [15] considered the two-dimensional model of a single phase of Tiwari–Das steady flow over a stretched and shrinking sheet during the analysis of a Casson-based nanofluid.

Researchers have attempted to incorporate numerous solid nanoparticles through various types of base fluids in response to the increasing requirements for heat transfer in several branches of the industry, as previously mentioned. As a result, a hybrid nanofluid, the newest type of nanofluid, was developed. According to Devi and Devi [16], combining a metal nanoparticle in a small quantity/nanotube with an oxide/suspension of metal nanoparticles in a base fluid will dramatically boost thermal properties. Common fluids usually have more than one solid particle, such as glycol, paraffin oil, water, vegetable oil, engine oil, kerosene, and ethylene. According to Huminic and Huminic [17], there are several significant applications of hybrid nanofluids. For example, these liquids have been used for heat transfer in exchangers of plate heat, micro channels, air-conditioning systems, etc. Radiation’s effects on the steady flow of a two-dimensional hybrid nanofluid on a nonlinear stretched/shrunk surface were investigated by Waini et al. [18]. Moreover, Waini et al. [19] studied the influence of transpiration on the steady flow of a two-dimensional hybrid nanofluid and heat transfer through a stretched/shrunk sheet with a uniform shear flow.

Many fields use rotational flow, such as engineering processes (e.g., rotating machinery and computer storage devices), electronic devices, extrusion of plastic sheets, and the centrifugal filtration process. Hayat et al. [20] investigated the Ag–CuO/water three-dimensional hybrid nanofluid’s steady flow in the presence of radiation, chemical reactions, and heat generation. It was discovered that a hybrid fluid has a superior rate of energy transfer compared toa regular nanofluid. When the rotation parameter is raised, the concentration profile increases. Furthermore, Hayat et al. [21] scrutinized a three-dimensional rotating hybrid nanofluid flow using boundary influence with radiation and partial slip. They came to the conclusion that the temperature profile improved as the rotation and radiation parameters were increased. Later, Anuaret al. [22] studied the hybrid nanofluid steady rotating flow for a stretched/shrunk surface under the influence of radiation. On hybrid nanofluids, numerous scholars have investigated the stretching/shrinking sheet and the rotating sheet in models with different considerations [23,24,25,26].

Magnetohydrodynamics (MHD) is a term that combines the words magneto (magnetic), hydro (fluids), and dynamics (motion). Magnetohydrodynamics is the study of the flow of electrically conducting fluids in the presence of a magnetic field. Since a magnetic field can occur anywhere in the universe, MHD phenomena can also occur when conducting fluids are present in natural phenomena. Engineers use MHD concepts in the project and design of an extensive range of applications in industries (e.g., heat exchanger generators and MHD pumps) [27]. So it is vital. Devi and Devi [16] numerically explored the two-dimensional magnetic –time-independent flow through a stretched sheet with suction and the Newtonian heating effect in a hybrid nanofluid. Furthermore, Devi and Devi [28], with Newtonian heating, expanded work to three-dimensional time-independent. They reported that a hybrid nanofluid transfers heat more quickly than a normal nanofluid. After that, Devi and Devi [29] examined the two-dimensional steady flow of a hybrid nanofluid over a stretched sheet. In a hybrid nanofluid, Aly and Pop [30] studied the two-dimensional steady flow over a stretched and shrunk plate using suction, the biot number, and MHD effects. Wainiet al. [31] explored the influence of hybrid nanoparticles on a fluid’s steady flow in an exponentially stretching/shrinking two-dimension allayer. It was found that suction effects and shrinking effects had dual solutions. Lund et al. [32] analyzed two-dimensional unsteady flow of a hybrid nanofluid on stretching and shrinking sheets under the influence of radiation and MHD. Teh and Asghar [33] studied the MHD hybrid nanofluid flow under the influence of Joule heating on a three-dimensional rotating stretching/shrinking surface. The act of creating heat by passing current through a conductor is known Joule heating, also known as ohmic heating, resistance heating, or resistive heating. Incandescent lightbulbs, resistance ovens, electric stoves, soldering irons, and cartridge heaters are all examples of Joule heating. Additionally, Khashi et al. [34] assessed the two-dimensional steady flow behavior of a hybrid $Cu-Al_{2}O_{3}$/water nanofluid associated with a radially stretched/shrunk sheet under the impact of Joule heating, MHD, and suction. Moreover, Yan et al. [35] explored the impact of Joule heating on electrically conducting hybrid nanofluid flow moving over an exponential surface.

Thermal radiation has a significant influence at high operating temperatures that cannot be overlooked. Radiation sensitivity is crucial in the design of a suitable method since several industrial processes take place at extremely high temperatures. It also plays a substantial role in a variety of industrial applications, such as glass processing, furnace construction, internal combustion engines, plasma physics, and spacecraft [36].Sreedevi et al. [37] investigated the two-dimensional unsteady flow of mass and heat transfer of a hybrid nanofluid on a stretched surface by radiation. Recently, Dero et al. [38] studied the numerical analysis of the $Cu+Al_{2}O_{3}/$water hybrid nanofluid in terms of cross-flow and stream wise under the influence of thermal radiation. Some study has been done on flow and thermal radiation. Some current publications can be found in these studies [39,40,41].

In the two-dimensional case, research on hybrid nanofluids under MHD, radiation, and Joule heating has been rigorous (see Waini et al. [18], Khashi’ie et al. [34], and Yan et al. [35]). According to the literature survey on previous studies, it is critical to address such a problem as three-dimensional flow since this setting enables more extensive and realistic real-world applications. The present study expanded on the work of Anuar et al. [22] by incorporating the influence of radiation, MHD, and Joule heating using the research of Tiwari and Das [42]. Therefore, for this study, a novel physical model of the influence of thermal radiation on the three-dimensional magnetized rotating flow of a hybrid nanofluid with Joule heating is developed. To reach high convective heat transfer efficiency, in the hybrid nanofluid measured in this study, alumina ($Al_{2}O_{3}$) and copper (Cu) nanoparticles are used. By suspending two nanoparticles, a hybrid nanofluid ($Al_{2}O_{3}-Cu$/water) is designed. Profiles of temperature and velocity for several values of volume fraction copper, magnetic, Eckert number, and radiation parameters of solid nanoparticles are examined in this study. Furthermore, the effect of solid nanoparticle copper against stretched/shrunk and suction parameters in terms of declined heat transfer and change in skin friction are also included in this study. The present numerical findings are compared to the results of prior investigations for comparison purposes. To the best of our knowledge, this model is different and new and no related article has been found in the literature.

## 2. Materials and Methods

### 2.1. Mathematical Formulation

**Figure 1:**
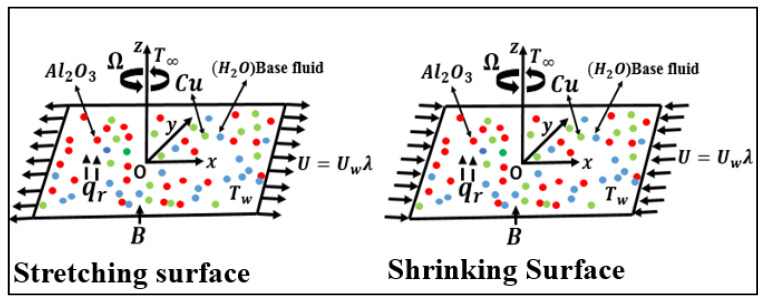
The physical model of a stretching/shrinking surface.

Three-dimensional hybrid nanofluid steady flow with heat transfer in stretched and shrunk sheets is shown in Figure 1, which has Cartesian coordinates with the x-axis and the y-axis measured in the z=0 plane. The fluid that occupies half of the area is at z≥0. In the x-direction, the surface is deemed to be stretched/shrunk only when velocity $U_{w}=ax$ and temperature is $T_{w}=T_{\infty }+T_{0}x^{2}$ [43]. The entire system rotates at a constant velocity Ω along a perpendicular to the surface z-axis. In the z-axis, there is a variable magnetic field B and radiation $q_{r}$. The surface of the hybrid nanofluid is constant and has ambient temperature $T_{\infty }$.$T_{w}$ is the wall temperature, and $T_{0}$ is the characteristic temperature. The mathematical governing equations help understand the physical model by indicating that the flow is incompressible, Newtonian, and laminar. Figure 1 shows that fluid stages and nanoparticles are considered in a thermal equilibrium state. They are distinctly tiny and uniform in shape, allowing the slip velocity among phases to be discounted.

The governing equation for a hybrid nanofluid is as follows [22]:

(1) $$u_{x}+v_{y}+w_{z}=0,$$

(2) $$uu_{x}+vu_{y}+wu_{z}-2\Omega v=\frac{\mu_{hnf}}{\rho_{hnf}}u_{zz}-\frac{\sigma_{hnf}}{\rho_{hnf}}B^{2}u,\;$$

(3) $$uv_{x}+vv_{y}+wv_{z}+2\Omega u=\frac{\mu_{hnf}}{\rho_{hnf}}v_{zz}-\frac{\sigma_{hnf}}{\rho_{hnf}}B^{2}v,\;$$

(4) $$uT_{x}+vT_{y}+wT_{z}=\frac{k_{hnf}}{{\left(\rho c_{p}\right)}_{hnf}}T_{zz}+\frac{\sigma_{hnf}}{{\left(\rho c_{p}\right)}_{hnf}}B^{2}\left(u^{2}+v^{2}\right)-\frac{1}{{\left(\rho c_{p}\right)}_{hnf}}q_{rz}\;,\;$$

The boundary conditions are

(5) $$\left\{\begin{array}{c} u=\lambda U_{w}\;\;,\;v=0\;\;\;,\;w=w_{w}\;\;,\;T=T_{w},\;as\;\;z=0,\\ u\to 0,\;\;\;\;\;\;\;\;\;\;\;v\to 0,\;\;\;\;\;\;\;T\to T_{\infty },\;\;as\;\;z\to \infty \end{array}\right.$$

The hybrid nanofluid velocity components u, v, and w are along the *x*-axis, the *y*-axis, and the *z*-axis, respectively. λ is the stretched/shrunk parameter such that λ<0 shows the shrinking surface, λ=0 represents the static surface, and λ>0 indicates the stretching sheet. T represents temperature. Hybrid nanofluid thermophysical properties were used to determine the above equation, as mentioned in Table 1 and Table 2. Alumina ($Al_{2}O_{3}$) nanoparticles are denoted by $\phi_{1}$, and copper (Cu) nanoparticles are indicated by $\phi_{2}$. It should be noted that the current model is used for only spherical nanoparticles because it gives better heat transfer performance. Moreover, $c_{p},\;\;\rho ,\;\;\mu ,\;\;\sigma$, and k correspond with specific heat capacity, density, dynamic viscosity, electrical conductivity, and thermal conductivity, respectively. Fluid, nanofluid, hybrid nanofluid, solid nanoparticles 1 ($Al_{2}O_{3}),$ and solid nanoparticles 2 (Cu) are denoted by subscripts f, nf, hnf, s1, and s2, respectively.

The similarity factors indicated below are employed in this example [44].

(6) $$u=axf^{\prime }\left(\eta \right),\;v=axg\left(\eta \right),w=-\sqrt{a\nu_{f}}f\left(\eta \right),\;\theta \left(\eta \right)=\frac{T-T_{\infty }}{T_{w}-T_{\infty }},\eta =z\sqrt{\frac{a}{\nu_{f}}}$$

Here, prime shows the differentiation for  η, a is a stretching constant, and $\nu_{f}$ is the kinematic viscosity. Although $w_{w}=-\sqrt{a\nu_{f}}S$, S stands for the injection/suction parameter. When S<0, the flow is injection, and when the S>0, the flow is suction.

Equation (1) is identically satisfied by inserting Equation (6) in Equations (2)–(5). Subsequently, Equations (2)–(5) are transformed into the following ODEs.

(7) $$\frac{\mu_{hnf}/\mu_{f}}{\rho_{hnf}/\rho_{f}}f^{\prime \prime \prime }-{\left(f^{\prime }\right)}^{2}+ff^{\prime \prime }+2\omega g-\frac{\sigma_{hnf}/\sigma_{f}}{\rho_{hnf}/\rho_{f}}Mf^{\prime }=0$$

(8) $$\frac{\mu_{hnf}/\mu_{f}}{\rho_{hnf}/\rho_{f}}g^{\prime \prime }+fg^{\prime }-f^{\prime }g-2\omega f^{\prime }-\frac{\sigma_{hnf}/\sigma_{f}}{\rho_{hnf}/\rho_{f}}Mg=0$$

(9) $$\frac{1}{Pr{\left(\rho c_{p}\right)}_{hnf}/{\left(\rho c_{p}\right)}_{f}}\left\{\frac{k_{hnf}}{k_{f}}+\frac{4Rd}{3}\right\}\theta^{\prime \prime }+f\theta^{\prime }-2f^{\prime }\theta +\frac{\sigma_{hnf}/\sigma_{f}}{{\left(\rho c_{p}\right)}_{hnf}/{\left(\rho c_{p}\right)}_{f}}MEc\left({f^{\prime }}^{2}+g^{2}\right)=0$$

Subject to conditions:

(10) $$\left\{\begin{array}{c} f\left(0\right)=S,\;\;\;\;g\left(0\right)=0,\;\;\;\;\;f^{\prime }\left(0\right)=\lambda ,\;\;\;\;\theta \left(0\right)=1\\ \;f\prime \left(\eta \right)\to 0,\;\;\;\;\;\theta \left(\eta \right)\to 0,\;\;\;\;\;\;g\left(\eta \right)\to 0\;\;\;\;as\;\;\;\eta \to \infty \end{array}\right.$$

where ω is the rotation, M the magnetic parameters, Ec is the Eckert number, $q_{r}$ is the radiative heat flux, and Pr is Prandtl number, which is described by:

(11) $$\omega =\frac{\Omega }{a},M=\frac{B^{2}\sigma_{f}}{a\rho_{f}},Ec=\frac{u_{w}^{2}}{(T_{w}-T_{\infty }){\left(c_{p}\right)}_{f}},q_{r}=-\frac{4\sigma_{1}}{3k^{1}}\frac{\partial T^{4}}{\partial y},Pr=\frac{{\left(\mu c_{p}\right)}_{f}}{k_{f}}$$

The skin friction coefficients $C_{fx}$ and $\;C_{fy}$ show the x-axis and the y-axis, respectively. The local Nusselt number $Nu_{x}$ is specified as:

(12) $$C_{fx}=\frac{\mu_{hnf}}{\rho_{f}U^{2}{}_{W}}{\left(u_{z}\right)}_{Z=0},\;\;\;C_{fy}=\frac{\mu_{hnf}}{\rho_{f}U^{2}{}_{W}}{\left(v_{z}\right)}_{Z=0},Nu_{x}=\frac{x}{k_{f}(T_{w}-T_{\infty })}\left[-k_{hnf}{\left(T_{z}\right)}_{z=0}+q_{r}{|}_{z=0}\right]$$

By using Equations (6) and (12), we get:

(13) $$\sqrt{Re_{x}}\;C_{fx}=\frac{\mu_{hnf}}{\mu_{f}}f^{\prime \prime }\left(0\right),\;\sqrt{Re_{x}}\;C_{fy}=\frac{\mu_{hnf}}{\mu_{f}}g^{\prime }\left(0\right),\frac{Nu_{x}}{\sqrt{Re_{x}}}=-\left[\frac{k_{hnf}}{k_{f}}+\frac{4}{3}Rd\right]\theta^{\prime }\left(0\right)$$

Here, $Re=\frac{U_{w}\;x}{\nu_{f}}$ is the local Reynolds number.

The physical properties of the solid nanoparticles and the base fluid(water) are presented in Table 2 [27,33].

### 2.2. Stability Analysis

The system of Equations (7)–(9) indicates that more than one numerical solution exists in a specific array of stretched/shrunk and suction parameters. Therefore, an analysis of stability is needed to determine which of the solutions is stable [45,46]. That being stated, the transformation requires the introduction of new similarity variables, which are defined as

(14) $$\left\{\begin{array}{c} =axf^{\prime }\left(\eta ,\tau \right),\;v=axg\left(\eta ,\tau \right),w=-\sqrt{a\nu_{f}}f\left(\eta ,\tau \right)\\ \;\theta \left(\eta ,\tau \right)=\frac{T-T_{\infty }}{T_{w}-T_{\infty }},\eta =z\sqrt{\frac{a}{\nu_{f}}},\tau =at \end{array}\right.$$

where τ represents the dimensionless time (t). When Equation (14) is substituted into the unsteady Equations (2)–(4), the following equations can be acquired.

(15) $$\frac{\mu_{hnf}/\mu_{f}}{\rho_{hnf}/\rho_{f}}f_{\eta \eta \eta }-{\left(f_{\eta }\right)}^{2}+ff_{\eta \eta }+2\omega g-\frac{\sigma_{hnf}/\sigma_{f}}{\rho_{hnf}/\rho_{f}}Mf_{\eta }-f_{\tau \eta }=0$$

(16) $$\frac{\mu_{hnf}/\mu_{f}}{\rho_{hnf}/\rho_{f}}g_{\eta \eta }+fg_{\eta }-f_{\eta }g-2\omega f_{\eta }-\frac{\sigma_{hnf}/\sigma_{f}}{\rho_{hnf}/\rho_{f}}Mg-g_{\tau }=0$$

(17) $$\frac{\left\{\frac{k_{hnf}}{k_{f}}+\frac{4Rd}{3}\right\}}{Pr{\left(\rho c_{p}\right)}_{hnf}/{\left(\rho c_{p}\right)}_{f}}\theta_{\eta \eta }+f\theta_{\eta }-2f_{\eta }\theta +\frac{\sigma_{hnf}/\sigma_{f}}{{\left(\rho c_{p}\right)}_{hnf}/{\left(\rho c_{p}\right)}_{f}}MEc\left({\left(f_{\eta }\right)}^{2}+g^{2}\right)-\theta_{\tau }=0$$

With the new boundary condition

(18) $$\left\{\begin{array}{c} f\left(0,\tau \right)=S,\;f^{\prime }\left(0,\tau \right)=\lambda ,\;g\left(0,\tau \right)=0,\theta \left(0,\tau \right)=1\;\;\;\;\;\\ f^{\prime }\left(\eta ,\tau \right)\;\to \;0,g\left(\eta ,\tau \right)\;\to \;0\;\;\theta \left(\eta ,\tau \right)\to 0,\;\;\;as\;\eta \to \infty \end{array}\right.$$

We use perturbed equations to verify the steady flow stability of $f\left(\eta \right)=f_{0}\left(\eta \right),\;\;g\left(\eta \right)=g_{0}\left(\eta \right)$, and $\theta \left(\eta \right)=\theta_{0}\left(\eta \right)$, which satisfy Equations (7)–(9).

(19) $$\left\{\begin{array}{c} f\left(\eta ,\tau \right)=f_{0}\left(\eta \right)+e^{-\varepsilon \tau }F\left(\eta ,\tau \right)\\ g\left(\eta ,\tau \right)=g_{0}\left(\eta \right)+e^{-\varepsilon \tau }G\left(\eta ,\tau \right)\\ \theta \left(\eta ,\tau \right)=\theta_{0}\left(\eta \right)+e^{-\varepsilon \tau }H\left(\eta ,\tau \right) \end{array}\right.$$

where $F\left(\eta ,\tau \right),\;G\left(\eta ,\tau \right)$, and $H\left(\eta ,\tau \right)$ are modest relative to $f_{0}\left(\eta \right),\;\;g_{0}\left(\eta \right),$ and $\theta_{0}\left(\eta \right)$, respectively, and ε is the eigenvalue parameter. The following linearized eigenvalue issue is generated by inserting Equation (19) into Equations (15)–(17), fixing τ→0 and simplifying the equations.

(20) $$\frac{\mu_{hnf}/\mu_{f}}{\rho_{hnf}/\rho_{f}}F_{0}^{\prime \prime \prime }-{\left(F_{0}^{\prime }\right)}^{2}+f_{0}F_{0}^{\prime \prime }+F_{0}f_{0}^{\prime \prime }+2\omega G_{0}-\frac{\sigma_{hnf}/\sigma_{f}}{\rho_{hnf}/\rho_{f}}MF_{0}^{\prime }+\varepsilon F_{0}^{\prime }=0$$

(21) $$\frac{\mu_{hnf}/\mu_{f}}{\rho_{hnf}/\rho_{f}}G_{0}^{\prime \prime }g_{0}^{\prime }F_{0}+G_{0}^{\prime }f_{0}-\left(f_{0}^{\prime }G_{0}+F_{0}^{\prime }g_{0}\right)-2\omega F_{0}^{\prime }-\frac{\sigma_{hnf}/\sigma_{f}}{\rho_{hnf}/\rho_{f}}MG_{0}+\varepsilon G_{0}=0$$

(22) $$\frac{\left\{\frac{k_{hnf}}{k_{f}}+\frac{4Rd}{3}\right\}}{Pr{\left(\rho c_{p}\right)}_{hnf}/{\left(\rho c_{p}\right)}_{f}}H_{0}^{\prime \prime }+\theta_{0}^{\prime }F_{0}+H_{0}^{\prime }f_{0}-2\left(f_{0}^{\prime }H_{0}+F_{0}^{\prime }\theta_{0}\right)+\frac{\sigma_{hnf}/\sigma_{f}}{{\left(\rho c_{p}\right)}_{hnf}/{\left(\rho c_{p}\right)}_{f}}2MEc\left({\left(f_{0}^{\prime }F_{0}^{\prime }\right)}^{2}+G_{0}g_{0}\right)+\varepsilon H_{0}=0$$

subject to boundary conditions

(23) $$\left\{\begin{array}{c} F_{0}\left(0\right)=0,\;F_{0}^{\prime }\left(0\right)=0,\;G_{0}\left(0\right)=0,H_{0}\left(0\right)=0\\ F_{0}^{\prime }\left(\eta \right)\;\to \;0,G_{0}\left(\eta \right)\;\to \;0\;\;H_{0}\left(\eta \right)\to 0,\;\;\;as\;\eta \to \infty \end{array}\right.$$

The boundary conditions stated in Equation (23) must be changed prior to resolving the problem of eigenvalue. Harris et al. [47] proposed relaxing and replacing one of the far-field conditions. So, in this research, relaxing $F_{0}^{\prime }\left(\infty \right)\to 0$ is impossible since it will be substituted by $F_{0}^{\prime \prime }\left(0\right)=1$, which can influence the other numerical solutions and boundary conditions. As a result, we selected $F_{0}^{\prime }\left(\infty \right)\to 0$ to be loosened and substituted with $F_{0}^{\prime \prime }\left(0\right)=1$. This allows the stability of the solution to be determined, where $\varepsilon_{1}>0$ indicates that the solution is stable, whereas $\varepsilon_{1}<0$ shows that the solution is unstable owing to the development of the disturbance of the solution.

## 3. Results and Discussion

The solution’s duality has been achieved in the figures by using various initial guesses for $f^{\prime \prime }\left(0\right)$, $g^{\prime }\left(0\right)$, and $\theta^{\prime }\left(-0\right)$, with the outcome that both velocities and temperature profiles satisfied the boundary condition η→∞ asymptotically. By using the software MATLAB bv4pc solver, we numerically solved nonlinear ODEs (Equations (7)–(9)) with the boundary equation (Equation (10)). The bvp4c solver was created by Jacek Kierzenka and Lawrence F. Shampine of Southern Methodist University, Texas [48]. The bvp4c solver is a finite difference method that uses the three-stage Lobatto IIIA algorithm to provide numerical solutions with fourth-order precision. Before starting to discuss the results of the current study, we have compared the coding of a numerical method to make sure that our computer code is working properly. First, to validate the coding of a numerical scheme in this study, the reduced skin friction $f^{\prime \prime }\left(0\right)$ and $g^{\prime }\left(0\right)$ for pure waterwhen $\phi_{1}=\phi_{2}=0,\;\;S=0$, M=Rd=Ec=0, and Pr=6.2 is compared with values issued by Nazar et al. [49] and Anuaret al. [22], as in Table 3, for stretching sheet  λ = 1. The findings are consistent with those of previous studies. To yield the wanted hybrid nanofluid, i.e., $Al_{2}O_{3}-Cu$/water, first, we show that alumina is isolated in the base fluid (water). After that, copper (Cu) is dispersed into the nanofluid $Al_{2}O_{3}$/water. Here, $\phi_{1}$ represents alumina ($Al_{2}O_{3})$ and $\phi_{2}$ represents copper (Cu).

**Figure 2:**
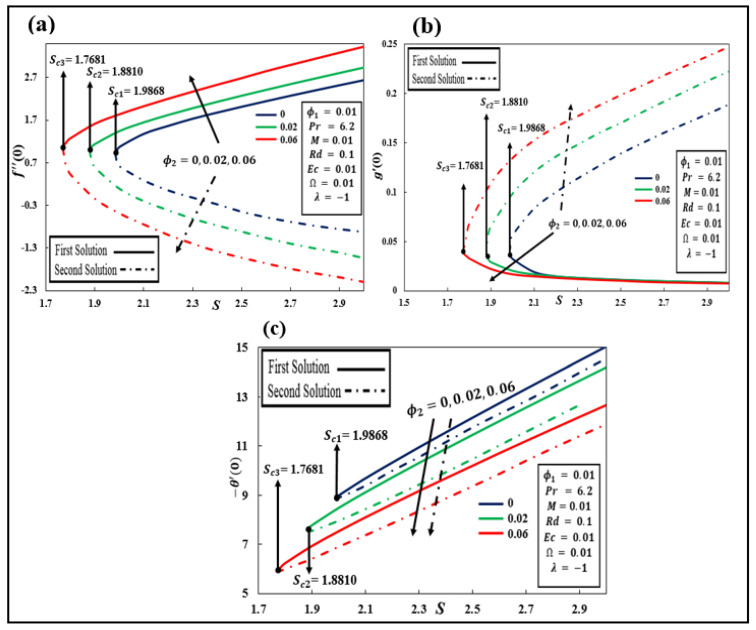
(**a**–**c**): Influence of $\phi_{2}\left(Cu\right)$ against suction $\left(S\right)$ on $f^{\prime \prime }\left(0\right)$, $g^{\prime }\left(0\right)$, and $-\theta^{\prime }\left(0\right)$.

The suction parameter ( S) of various values with the variation in $f^{\prime \prime }\left(0\right)$, $g^{\prime }\left(0\right)$, and $–\theta^{\prime }\left(0\right)$ with three values of solid nanoparticle volume fraction copper $\phi_{2}$ispresented in Figure 2a–c for λ=−1 (shrinking sheet) with the occurrence of different parameter values $\phi_{1}=0.01,\;Pr=6.2,\;\;M=0.01,\;\;Rd=0.1,\;\;Ec=0.01,\;$and Ω=0.01. The range of solid nanoparticles $\phi_{2}$ is 0 to 0.06. Here, $i=1,\;2,\;3.\;S_{ci}$ shows the critical point at the suction parameter where both solutions meet each other. It can be shown that when $S<S_{ci}$, no solution exists. Furthermore, the values of $S_{ci}\;$ = 1.9868, 1.8810, and 1.7681 are relative critical points of $\phi_{2}=0.0.02,$ and 0.06, respectively. It is worth revealing here that when $\phi_{2}=0$, it is purely $Al_{2}O_{3\;}$ water-based nanofluid and $S_{c1}=1.9868$. After that, 2% of $\phi_{2}$ is added and $\;S_{c2}=1.8810$ is achieved. Moreover, the value of $S_{c3}=1.7681$ appears to rise as 6% of the solid volume fraction of $\phi_{2}$ is added to the hybrid nanofluid.The boundary layer separation extends as the value of $\phi_{2}$ increases. From Figure 2a, in the first solution, when values of $\phi_{2}$ increase, the reduced skin friction $f^{\prime \prime }\left(0\right)$ increases and on the other side, the values of $f^{\prime \prime }\left(0\right)$ decrease in the second solution. In Figure 2b, the first solution declines and the second solution of $g^{\prime }\left(0\right)$ is improved when the values of $\phi_{2}$ increase. As can be seen in Figure 2c, when the value of $\phi_{2}$ rises, the magnitude of $-\theta^{\prime }\left(0\right)$ is also decreased in both first and second solutions.

**Figure 3:**
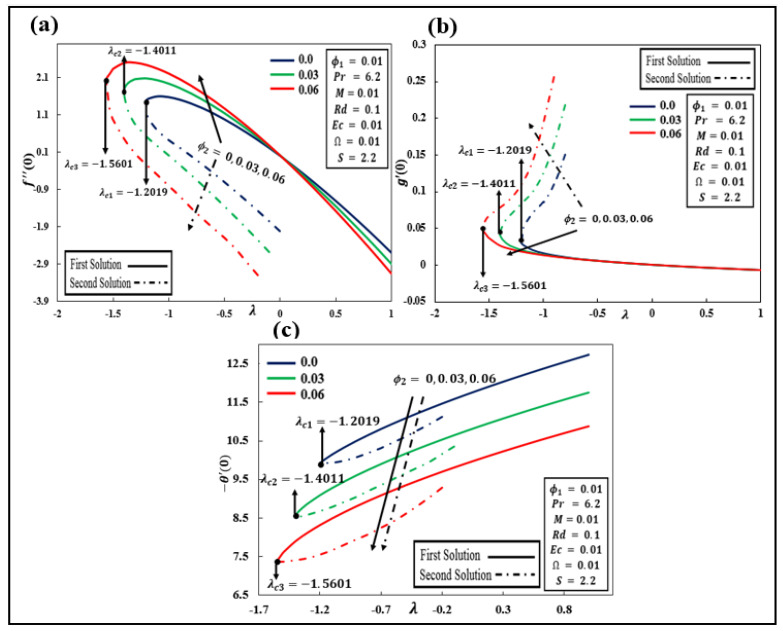
(**a**–**c**): Influence of $\phi_{2}\left(Cu\right)$ against stretching/shrinking $\left(\lambda \right)$ on $f^{\prime \prime }\left(0\right)$, $g^{\prime }\left(0\right)$, and $-\theta^{\prime }\left(0\right)$.

Due to the existence of multiple solutions, we investigated which of the governing parameters can play a role in the formation of dual solutions. Against varying values of solid nanoparticles, Figure 3a–c demonstrates volume fraction $\phi_{2}=0.0,\;0.03,\;\mathrm{and}\;0.06$ along with reduced skin friction $\;f^{\prime \prime }\left(0\right)$ and $g^{\prime }\left(0\right)$ and reduced heat transfer rate $-\theta^{\prime }\left(0\right)$ through the presence of parameters $\phi_{1}=0.01,\;\;Pr=6.2,\;\;M=0.01,\;Rd=0.1,\;\;Ec=0.01,\Omega =0.01,$ and S=2.2. For shrinking sheet λ≤−1, we can understand that the presence of non-unique solutions is notable. When i=1, 2, 3,$\lambda_{ci}$ represents the critical point at which both solutions meet at the shrinking sheet parameter. It can be shown that no solution exists when $\lambda <\lambda_{ci}$ but a unique solution is observed when  λ>−1. Beyond these critical values, the boundary layer estimate is no longer defensible. Furthermore, the values of $\lambda_{c1}=-1.2019$,$\;\lambda_{c2}=-1.4011$, and $\lambda_{c3}=-1.5601$ are the corresponding critical points of $\phi_{2}=0.0,\;0.03,\;$and 0.06, respectively. This is proof that increasing the value of $\phi_{2}$ can cause the boundary layer separation to be delayed. Figure 3a shows that in the first solution, the value of $f^{\prime \prime }\left(0\right)$ increases when λ≤0 and decreases when λ>0, while the second solution declines after the increment of $\phi_{2}$. In Figure 3b, when the influence of $\phi_{2}$ improves, the value of $g^{\prime }\left(0\right)$ decreases in the first and increases in the second solution. In Figure 3c, $-\theta^{\prime }\left(0\right)$ declines in both solutions when $\phi_{2}$ increases. Similar findings can be seen in Anuar et al. [22,26].

**Figure 4:**
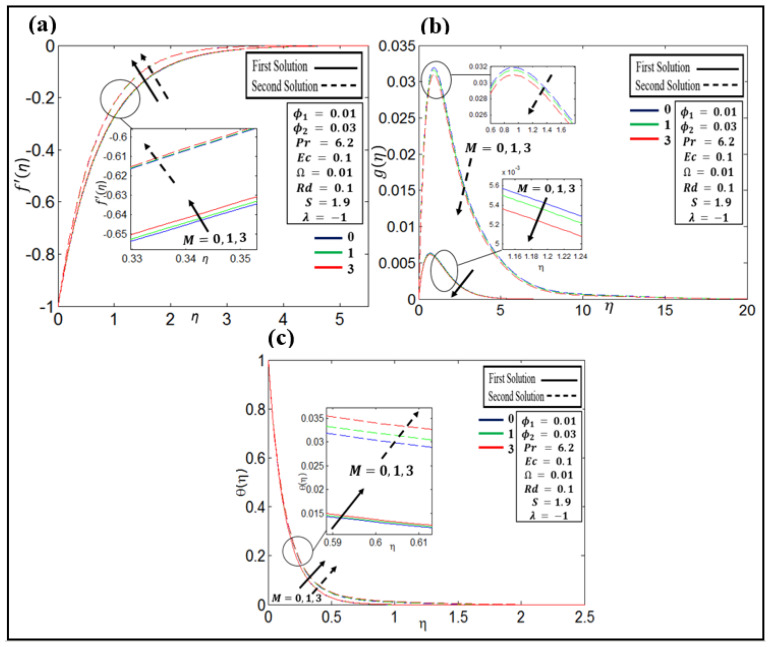
(**a**–**c**) Profiles of velocity $f^{\prime }\left(\eta \right)$ , $g\left(\eta \right)$, and temperature $\theta \left(\eta \right)$ for different  M.

Figure 4a–c depicts different values of M= 0, 1, and 3 when the velocity and temperature profiles are $\left(f^{\prime }\left(\eta \right),g\left(\eta \right)\right)$ and $\theta \left(\eta \right),$ respectively, under different parameters, such as $\phi_{1}=0.01,\;\;\phi_{2}=0.03,\;\;Pr=6.2,\;S=1.9,\;\;Rd=Ec=0.1,\;\;\Omega =0.01,$ and λ=−1. Figure 4a illustrates that $f^{\prime }\left(\eta \right)$ profiles decrease with an upsurged value of M in both (first and second) solutions due to the high drag force created by the magnetic number. The effect of M on $g\left(\eta \right)$ profiles is shown in Figure 4b. It is observed that $g\left(\eta \right)$ profiles reduce with an upsurged value of M in both solutions. Figure 4c shows that $\theta \left(\eta \right)$ increases as the value of M increases in both solutions. As a consequence, the influence of heat transfer is increased as M increases. The Lorentz force, which is caused by the magnetic field, makes the rate of transfer less resistant [49].

**Figure 5:**
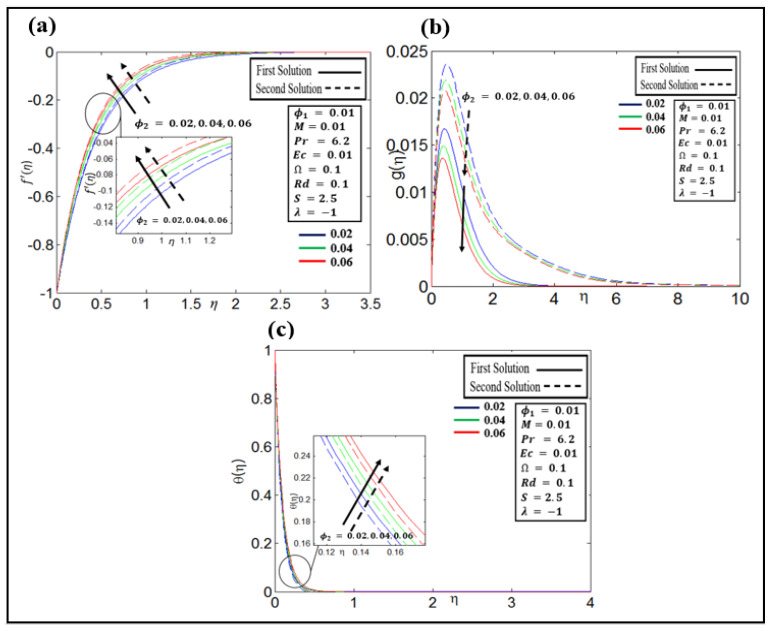
(**a**–**c**): Profiles of velocity $f^{\prime }\left(\eta \right)$, $g\left(\eta \right)$, and temperature $\theta \left(\eta \right)$ for different $\phi_{2}$.

The plots of the velocity profile $\left(f^{\prime }\left(\eta \right),g\left(\eta \right)\right)$ and the temperature profile $\theta \left(\eta \right)$ against solid nanoparticle volume fractions $\phi_{2}=0.02,\;0.04,\;$and 0.06 of copper are portrayed in Figure 5a–c, respectively. Figure 5a shows that in thefirst and second solutions, the fluid velocity $f^{\prime }\left(\eta \right)$ decreases when the value of $\phi_{2}$ is enhanced. From Figure 5b, the $g\left(\eta \right)$ profile in both solutions declines with a growing value of $\phi_{2}$. Figure 5c show that in the first and second solutions, the temperature profile $\theta \left(\eta \right)$ increases when the value of $\phi_{2}$ is enhanced.

**Figure 6:**
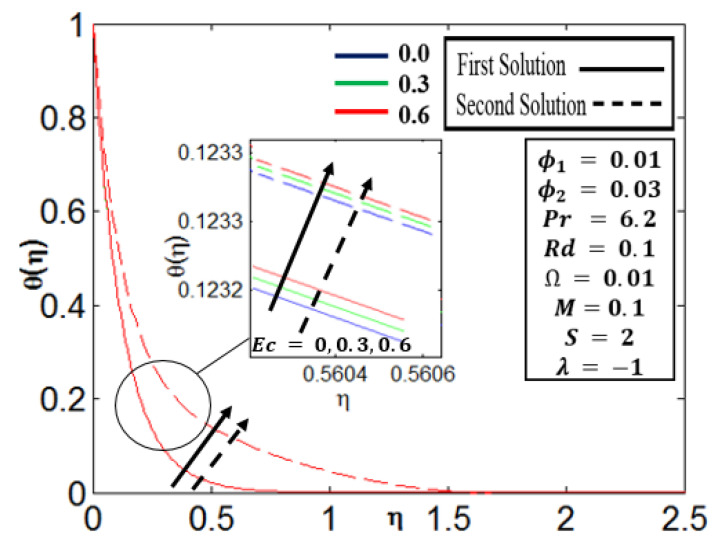
Profile of $\theta \left(\eta \right)$ for different Ec parameters.

Figure 6 illustrates the value of Ec at a different point for the temperature profile $\theta \;\left(\eta \right)$. In the first and second solutions, the values of Ec increase, causing $\theta \;\left(\eta \right)$ to rise as well. The intensity of heat transfer rises as the value of Ec grows due to the increasing heat created by Joule heating. It is worth noting that the influence of the increasing values of Ec on $\left(f^{\prime }\left(\eta \right),g\left(\eta \right)\right)$ is not important because, as seen in Table 4, the values remain the same for the increasing values of  Ec. Some variations in the value of Ec are provided in Table 4.

**Figure 7:**
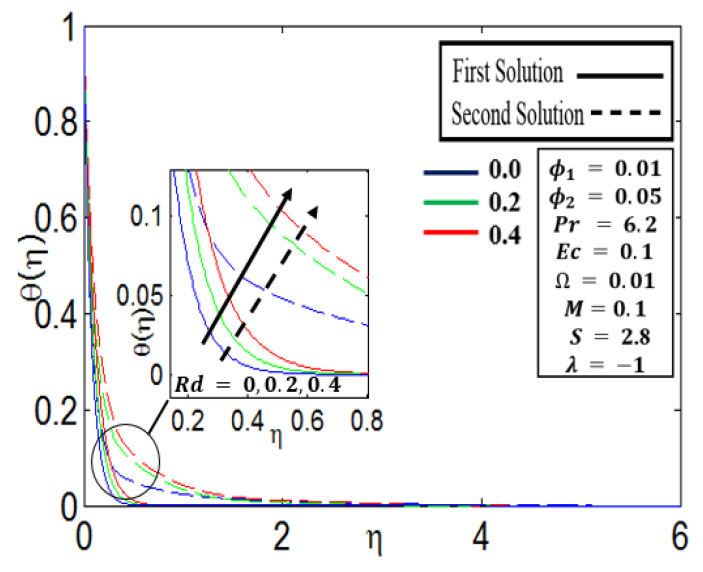
Profile of $\theta \left(\eta \right)$ for different Rd parameters.

Figure 7 shows the various values of radiation parameter Rd=0.0, 0.2, and 0.4 for the temperature profile $\theta \;\left(\eta \right)$. The radiation parameter Rd only exists in Equation (9). As a result, the values of Rd have no influence on the velocity profile since they are uncoupled from the momentum Equations (7) and (8).The thermal boundary layer thickness constantly rises in the first and second solutions with a growing value of  Rd, which means that higher Rd values result in a reduced temperature gradient at the surface.Due to the existence of high radiation, a big quantity of heat energy is produced in the system, which causes the temperature to increase and implies that the temperature of the fluid $\theta \;\left(\eta \right)$ rises.

Because dual solutions are obvious in the data, we ran a stability analysis to assess the solution’s practicability. Based on our findings, we determined that only the first option is stable and viable for use as guidance in real-world applications. This claim is supported by tabulation (see Table 5), which shows that the solution is stable when the executed minimum eigenvalues are positive and non-stable otherwise.

## 4. Conclusions

In this study, a three-dimensional $Al_{2}O_{3}-Cu$/water MHD hybrid nanofluid with heat transfer rotating flow on a linear stretched/shrunk surface underthe effect of radiation and Joule heating has been investigated through a bvp4c solver on the MATLAB computing platform. This research concentrates on the behavior of $f^{\prime \prime }\left(0\right)$, $g^{\prime }\left(0\right),-\theta^{\prime }\left(0\right),\;f^{\prime }\left(\eta \right),g\left(\eta \right),$ and $\theta \left(\eta \right)$ underthe effect of suction/injection, MHD, radiation, and Joule heating on the above-stated hybrid nanofluid flow. The following are the key conclusions of the present investigation:

1. The outcomes of Equations (2)–(5) are non-unique when λ≤−1 and unique when λ>1. No solution exists when $\lambda <\lambda_{ci}$ is noticed for a shrinking sheet.
2. The presence of solutions is based on the values of the suction parameter for assumed $\phi_{2}$ parameter values.
3. The intended model displays good heat transfer performance when nanoparticle sizes are less than 10%.
4. The temperature and velocity profiles of the boundary layer can be changed by applying nanoparticles in the hybrid nanofluid.
5. The profile $g\left(\eta \right)$ is reduced when the magnetic parameter is enhanced.
6. Ec and Rd values are directly proportional to temperature profiles in both solutions.
7. The first solution is a stable solution.

## Figures and Tables

**Table 1:** Thermophysical properties of a hybrid nanofluid [33].

Names	Properties
Dynamic viscosity	μhnf=μf1−ϕ1521−ϕ252
Density	$\rho_{hnf}=\left(1-\phi_{2}\right)\left[\left(1-\phi_{1}\right)\rho_{f}+\phi_{1}\rho_{s1}\right]+\phi_{2}\rho_{s2}$
Thermal conductivity	$k_{hnf}=\frac{k_{s2}+2k_{nf}-2\phi_{2}\left(k_{nf}-k_{s2}\right)}{k_{s2}+2k_{nf}+\phi_{2}\left(k_{nf}-k_{s2}\right)}\times \left(k_{nf}\right)$ $\mathrm{where}\;k_{nf}=\frac{k_{s1}+2k_{f}-2\phi_{1}\left(k_{f}-k_{s1}\right)}{k_{s1}+2k_{f}+\phi_{1}\left(k_{f}-k_{s1}\right)}\times \left(k_{f}\right)$
Heat capacity	${\left(\rho c_{p}\right)}_{hnf}=\left(1-\phi_{2}\right)\left[\left(1-\phi_{1}\right){\left(\rho c_{p}\right)}_{f}+\phi_{1}{\left(\rho c_{p}\right)}_{s1}\right]+\phi_{2}{\left(\rho c_{p}\right)}_{s2}$
Electrical conductivity	$\sigma_{hnf}=\frac{\sigma_{2}+2\sigma_{nf}-2\phi_{2}\left(\sigma_{nf}-\sigma_{2}\right)}{\sigma_{2}+2\sigma_{nf}+\phi_{2}\left(\sigma_{nf}-\sigma_{2}\right)}\times \left(\sigma_{nf}\right)$ $\mathrm{where}\;\left(\sigma_{nf}\right)=\frac{\sigma_{1}+2\sigma_{f}-2\phi_{1}\left(\sigma_{f}-\sigma_{1}\right)}{\sigma_{1}+2\sigma_{f}+\phi_{1}\left(\sigma_{f}-\sigma_{1}\right)}\times \left(\sigma_{f}\right)$

**Table 2:** Solid nanoparticles and base fluid(water) thermophysical properties.

Fluids	ρ (kg/m^3^)	σ Sm	$c_{p}\;(J/\mathrm{kg}\;K)$	*k* (W/m K)	Pr
Copper (*Cu*)	8933	$5.96\;\times \;10^{7}$	385	400	-
$\mathrm{Alumina}\;(Al_{2}O_{3})$	3970	$3.69\;\times \;10^{7}$	765	40	-
$\mathrm{Water}\;(H_{2}O$)	997.1	0.05	4179	0.613	6.2

**Table 3:** The comparison results of rotation parameter ω at different values when$\;\phi_{1}=\phi_{2}=0$, λ=1,  S=0,  M= Rd=Ec=0, and Pr=6.2.

	Nazar et al. [49]		Anuar et al. [22]		Current Results	
ω	$f^{\prime \prime }\left(0\right)$	$g^{\prime }\left(0\right)$	$f^{\prime \prime }\left(0\right)$	$g^{\prime }\left(0\right)$	$f^{\prime \prime }\left(0\right)$	$g^{\prime }\left(0\right)$
0.5	−1.1384	−0.5128	−1.13838	−0.51276	−1.138374	−0.512760
1.0	−1.3250	−0.8371	−1.35503	−0.83710	−1.325028	−0.837098
2.0	−1.6523	−1.2873	−1.65235	−1.28726	−1.65235	−1.287258

**Table 4:** The results of the Eckert number Ec  when $\phi_{1}=0.01$, $\phi_{2}=0.03$, S=2, ω=0.01, M = Rd=0.1, λ=−1, and Pr=6.2.

Ec	$-\theta^{\prime }\left(0\right)$
0	8.247357 (8.476272)
0.3	8.247162 (8.476187)
0.6	8.246967 (8.476103)

Note: The values inside the brackets represent the values of the second solution.

**Table 5:** The results of the suction parameter S at different values of $\phi_{2}$ when $\phi_{1}=0.01,\;\;\omega =0.01,\;\;M=0.01,\;\;Rd=0.1,\;\;Ec=0.01,\;\mathrm{and}\;\lambda =-1.$

		$\varepsilon_{1}$	
$\phi_{2}$	S	First Solution	Second Solution
0	3	0.2042	−0.2233
	2.5	0.1584	−0.2093
	2	0.0806	−0.1003
	1.9868	0.0071	−0.0251
0.02	3	0.8668	−0.8978
	2.8	0.7704	−0.7929
	2.6	0.5954	−0.5988
	2.4	0.3723	−0.4012
	2.2	0.1912	−0.0853
	1.8810	0.0108	−0.0071
0.06	3	1.1380	−1.0836
	2.8	1.0230	−0.9651
	2.6	0.8186	−0.7554
	2.4	0.6591	−0.5691
	2.2	0.3532	−0.2856
	2	0.0993	−0.1003
	1.7681	0.0080	−0.0006

## Data Availability

The data presented in this study are available on request from the corresponding author.
